# Curriculum Innovations: A Social Media–Based Educational Curriculum Improves Knowledge for Trainees in Neurocritical Care: Results of a Stratified Randomized Study

**DOI:** 10.1212/NE9.0000000000200087

**Published:** 2023-08-02

**Authors:** Ronald Alvarado-Dyer, Faddi G. Saleh Velez, Hera A. Kamdar, Naomi Niznick, Elizabeth Carroll, Carlos Castillo-Pinto, Melvin Parasram, Dearbhla Kelly, Shweta Goswami, Mikel S. Ehntholt, Neha S. Dangayach, Marc-Alain Babi, Ahmad Riad Ramadan, Christos Lazaridis, Catherine Albin, Nicholas A. Morris

**Affiliations:** From the Department of Neurology (R.A.-D., C.L.), University of Chicago Medical Center, University of Chicago, IL; Department of Neurology (F.G.S.V.), The University of Oklahoma Health Sciences Center, Oklahoma City; Department of Neurology (H.A.K.), Massachusetts General Hospital and Brigham and Women Hospital, Harvard Medical School, Boston; Department of Medicine (Neurology; Critical Care) (N.N.), The Ottawa Hospital, Ontario, Canada; Department of Neurology (E.C.), Columbia University Medical Center; Department of Neurology (E.C.), Cornell University School of Medicine, New York, NY; Department of Neurology (C.C.-P.), Seattle Children's Hospital, University of Washington, Seattle; Department of Neurology (M.P.), Yale School of Medicine, New Haven, CT; Department of Intensive Care Medicine (D.K.), St. James's Hospital, Dublin, and Global Brain Health Institute, Trinity College Dublin, Ireland; Department of Neurology (S.G.), University of Florida, Gainesville; Department of Neurology (M.S.E.), University of Pennsylvania, Philadelphia, PA; Department of Neurology (N.S.D.), Icahn School of Medicine at Mount Sinai, New York, NY; Department of Neurology and Neurosurgery (M.-A.B.), Neurological Institute, Cleveland Clinic Foundation (Florida), Port St. Lucie; Department of Neurology (A.R.R.), Henry Ford Health, Detroit, MI; Department of Neurology and Neurosurgery (C.A.), Emory University School of Medicine, Atlanta, GA; and Department of Neurology (N.A.M.), Program in Trauma, University of Maryland School of Medicine, Baltimore.

## Abstract

**Introduction and Problem Statement:**

The Neurocritical Care (NCC) Society Resident and Fellow Task Force's NEURON study concluded that learners had significant concerns regarding the need for educational improvement in NCC. To address these shortcomings, we identified the lack of an educational curriculum for trainees in NCC and developed a Twitter-based educational curriculum for trainees to improve knowledge in NCC.

**Objectives:**

The objectives of this study were to describe the pathophysiology, delineate a systematic diagnostic approach, and apply evidence-based strategies in the management of diseases in NCC.

**Methods and Curriculum Description:**

Ten trainees developed a Tweetorial (educational content available on Twitter)–based curriculum, with individual review by at least 2 NCC faculty. Learners were recruited through Twitter and randomized to 1 of 2 groups in a wait-list control prospective study. Group 1 completed the curriculum in the first 6 months of the 2021–2022 academic year, and group 2 completed the curriculum in the second half. Tweetorials were posted weekly on a private Twitter account only available to the active learner group. Learners were assessed by a multiple-choice format test (written by the trainees and reviewed by faculty) at 3 time points: before the first Tweetorial was released (preeducational curriculum assessment), after group 1 completed all tutorials and before group 2 started the curriculum (assessment 1), and after both groups finished (assessment 2). The primary outcome was the mean score on the second and third assessments.

**Results and Assessment Data:**

One hundred forty-six learners were assigned to group 1 or 2 using stratified block randomization including 99 (68%) Neurology residents, 81 (55%) US-based. Each group was composed of 73 participants. A total of 20 Tweetorials were published on a private Twitter account (@NeurocriticalE). Completed assessments were obtained from 100, 32, and 18 learners for the pre-educational curriculum assessment, assessment 1, and assessment 2, respectively. Group 1 and group 2 performed similarly in the pre-educational curriculum assessment. A potential for knowledge improvement was observed in group 1 at assessments 1 and 2 when compared with the learner group 2. Group 1 had more impressions, engagements, likes, URL clicks, and media views.

**Discussion and Lessons Learned:**

Although there was some learner attrition, our study demonstrates that social media can effectively deliver educational content and engage a diverse group of trainees around the globe.

## Introduction and Problem Statement

Physician trainees and advanced practice providers (APPs) often provide frontline care to critically ill neurologic and neurosurgical patients in the neurologic intensive care unit (NICU). Despite the establishment of neurocritical care (NCC) units (NCCUs) in academic centers across the United States, a 2012 survey of neurology residency directors revealed that only 56% of neurology residency programs have a dedicated NCCU rotation, and even across these programs, formal training in NCC is highly variable.^1,2^ When the Neurocritical Care Society Resident and Fellow Task Force's NEURON study surveyed neurology residents across the United States and 1 international NCC training program, they found that residents had significant concerns regarding the need for educational improvement within the NCCU.^3^ Given the demanding nature of the field, complex patient care, and varying call schedules, there is also limited time for and engagement with formal NCC didactics. We posited that an on-demand, asynchronous learning solution was needed to address educational gaps broadly across NCCUs. Thus, we developed a novel Twitter-based educational curriculum for trainees and APPs to improve their knowledge in NCC.

Social media has emerged as a relevant and growing tool for medical education. Twitter offers a rich interactive platform for disseminating educational content through Tweetorials, which are collections of threaded or consecutive tweets aimed at teaching users who engage with them.^4^ Furthermore, via Twitter analytics, Twitter also offers the possibility of gauging the interaction across followers with every individual tweet, which provides valuable insight regarding the interaction between users and the educational content delivered via Tweetorials. Tweetorials offer several features that are well adapted to adult learners including asynchronous learning and distilled points and require only a short investment of time to read for independent learning. As such, these microteaching posts are perfectly adapted “chalk talks” that can reach thousands of Twitter users. The coronavirus disease 2019 pandemic accelerated the adoption of social media platforms such as Twitter, leveraging its independent, asynchronous, interactive, and democratic features that flatten traditional educational hierarchies.^5^

Despite its growing popularity, a gap exists in showing evidence-based support for the educational efficacy of this newly embraced platform, particularly within NCC. In this study, we aimed to develop a social media–based NCC educational curriculum for trainees and APPs with the goal of improving knowledge in NCC disorders and assess the feasibility and efficacy of a Tweetorial-based educational curriculum.

## Curriculum Objectives

1. To describe the pathophysiology of diverse conditions in NCC (Table 1).
2. To delineate a systematic approach in the diagnosis of NCC disorders through case-based learning.
3. To apply evidence-based strategies in the management of diseases in NCC.

**Table 1:**
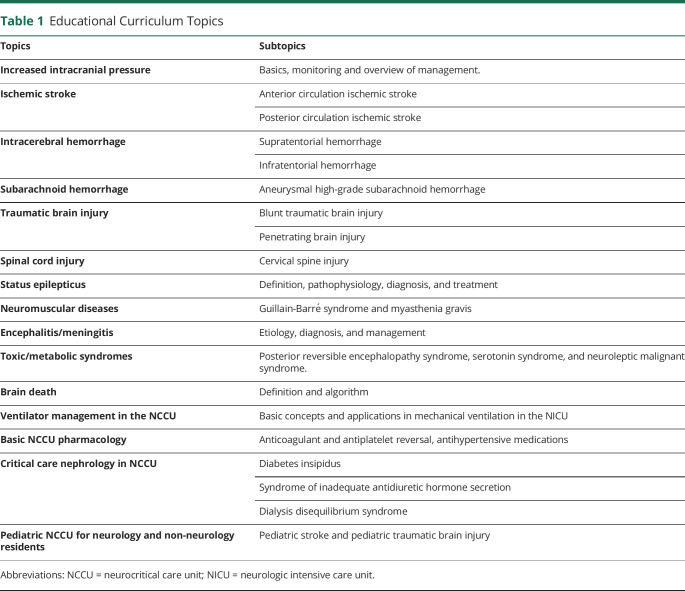
Educational Curriculum Topics

We developed and delivered a Twitter-based NCC educational curriculum to all the registered participants.

## Methods and Curriculum Description

### Curriculum Design

We developed a wait-list control educational curriculum among the recruited participants. The educational curriculum was staggered. The participants randomized to group 1 had the curriculum made available to them in the first half of the 2021–2022 academic year. The participants randomized to group 2 had the curriculum made available to them in the second half of the academic year. The educational curriculum was designed to be delivered over 5–6 months.

### Participant's Recruitment

The study was advertised through the author's Twitter accounts during the enrollment period. Trainees and APPs interested in participating in the educational curriculum completed an initial survey (distributed through Research Electronic Data Capture [REDCap]), which contained basic demographic information, current level of training, location of training, interest in NCC, prior rotation in an NCCU, if so, the duration of the rotation, and whether they would like to participate in the educational curriculum.

### Curriculum Content: Tweetorials

Ten trainees (8 NCC fellows, 1 vascular neurology fellow, and 1 pediatric neurology fellow) developed the curriculum. Each trainee wrote at least 1 case-based Tweetorial on an assigned topic that was reviewed by at least 2 of 6 NCC faculty from different academic institutions across the United States. Tweetorials were posted on a private Twitter account (@NeurocriticalE) on a weekly basis throughout the duration of the curriculum for each learner group. Each Tweetorial consisted of the following: a brief case presentation, initial NCC management, several links to scientific references for further review, key points of the pathology and management, and at least 1 poll.

### Educational Curriculum Outline/Syllabus

The curriculum was based on the published core curriculum and competencies for advanced training in neurologic intensive care (Table 1).^6^

### Curriculum Learning Outcomes: Assessments

In addition to authoring tweetorials, the participating fellows also collaborated in authoring a knowledge assessment test with a total of 65 multiple-choice questions on the covered topics. Each question was approved and verified by 6 board-certified neurointensivists prior to being included (same 6 neurointensivists who reviewed the Tweetorials). Two of the neurointensivists (N.A.M. and C.L.) were directly assigned by the main investigator (R.A.-D.). The other 4 (N.S.D., M.-A.B., A.R.R., C.A.) were recruited through Twitter. A total of 5 questions were discarded. Any question-related discrepancy was resolved by N.A.M. In the process of creating the assessments, we randomly sorted the questions into three 20-question assessments. All the tests were delivered via REDCap to each participant.

Before the initiation of the educational curriculum, all participants were expected to complete a 20-question pre-educational curriculum assessment to gauge baseline knowledge. Assessment 1 was expected to be completed by all participants after group 1 had finished the curriculum (but before group 2 has started the curriculum), and assessment 2 was expected to be completed by all participants after both learner groups completed the curriculum. The primary learning outcome of our curriculum was the mean score on the second and third assessments. The wait-list design controlled for the expected learning or maturation effect that takes place during a training program.

### Twitter Metrics

The Twitter analytics website^7^ was used to gather Twitter metrics after each learner group finished the educational curriculum. We collected the number of impressions, engagements, likes, URL clicks, and media views of all 20 published Tweetorials per learner group. Impressions were defined as the number of times users saw a Tweet on Twitter. Engagements were the number of times a user interacted with a Tweet (clicked anywhere on the Tweet). Likes indicated the number of times a user liked a Tweet. URL clicks referred to the number of times a user clicked on a website link within a Tweet. Media views denoted the number of times a user interacted with images and videos in a Tweet.

### Statistical Analysis

We reported participant's tests scores and Twitter metrics (impressions, engagements, likes, and social media views) using standard descriptive statistics (e.g., mean and SD for continuous normally distributed data and median and interquartile range for non-normally distributed data). We compared participants' tests scores, as well as group 1 and 2 Twitter metrics using student unpaired *t* test. Differences were considered significant with a *p* value <0.05. All analyses were performed using Stata/MP (version 15.0; StataCorp., College Station, TX) and photon 3 software. Only complete cases were analyzed at each time point. There was no intention-to-treat analysis, and there was no data imputation.

### Standard Protocol Approvals, Registrations, and Patient Consents

The study was reviewed and approved by The University of Chicago Institutional Review Board. A waiver of signed informed consent was granted. Through an initial REDCap survey, all the participants were provided an opportunity to agree or decline to participate in the educational curriculum.

### Data Availability

Data will be made available through request directed to the corresponding author.

## Results

### Participants

**Figure 1:**
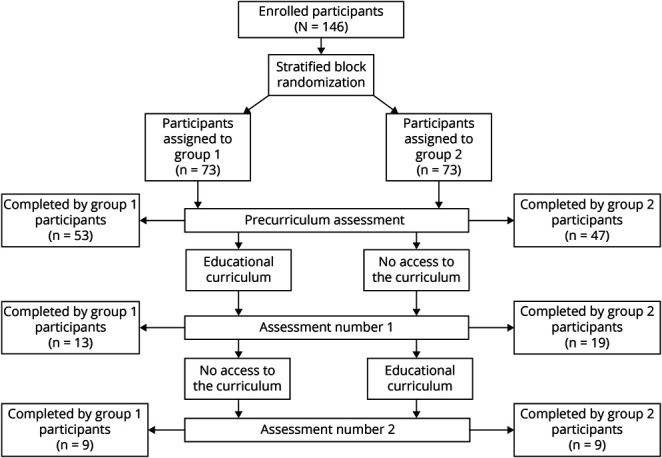
Educational Curriculum's Flowchart

A total of 146 learners participated (Figure 1). This included 99 (68%) neurology trainees and 45 (31%) trainees from other specialties such as anesthesia, child neurology, internal medicine, neurosurgery, emergency medicine, and pediatrics. Two participants (1%) were APPs. Of them, 81 (55%) were US-based, while 65 (45%) were non-US-based. One hundred (69%) had a dedicated NCCU at their institution. Ninety-two (63%) were considering pursuing an NCC fellowship. When comparing both groups, there was no statistically significant difference regarding the learners' mean age, sex (self-identified in the first REDCap survey), interest in pursuing an NCC fellowship, and participants who had an NCCU at their institution. Group 1 was composed of 57 (78%) neurology trainees, while group 2 had 42 (58%) neurology trainees. Similarly, group 1 had more US-based trainees when compared with group 2 (47 [64%] vs 34 [47%]) (Table 2).

**Table 2:**
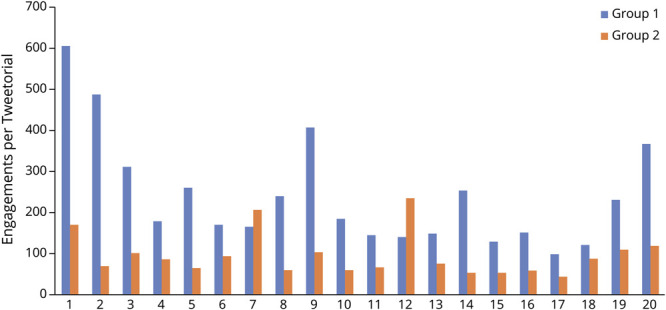
Participant Characteristics^a^

### Assessments

One hundred learners completed the pre-educational curriculum assessment. Thirty-two learners completed assessment 1, and 18 learners completed assessment 2. Both learner groups performed similarly in the pre-educational curriculum assessment. A potential for knowledge improvement was observed in group 1 at assessments 1 and 2 when compared with that in learner group 2.

### Twitter Metrics

**Figure 2:**
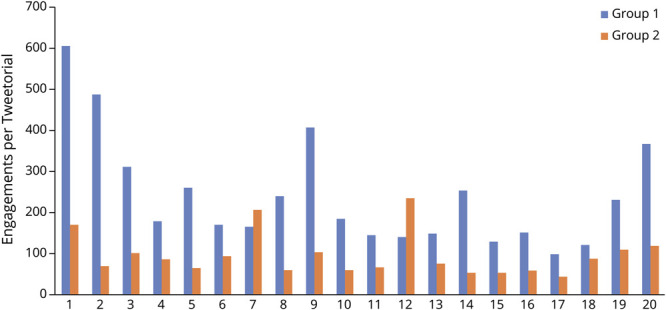
Engagements per Tweetorial

Learner group 1 had overall more impressions, engagements, likes, URL clicks, and media views than group 2 (Table 3). As summarized in eTable 3 (links.lww.com/NE9/A39) and presented in Figure 2, group 1 had consistently higher engagements throughout the curriculum.

**Table 3:**
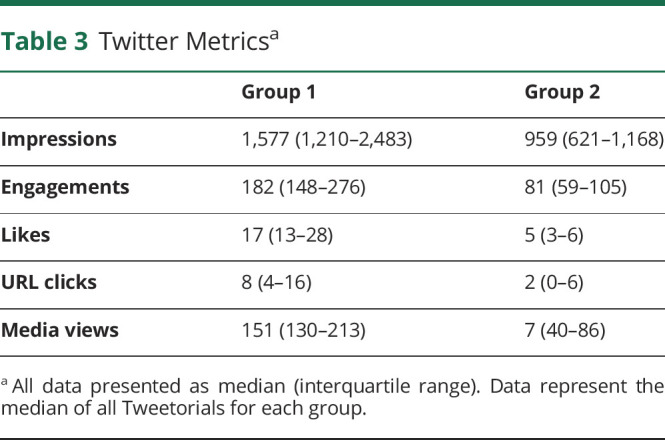
Twitter Metrics^a^

## Discussion and Lessons Learned

In this novel Tweetorial-based educational curriculum, we found out the following: (1) our Tweetorial-based education was able to reach learners with different backgrounds around the globe but lacked sustained curricular engagement; (2) the preliminary evaluation of our educational curriculum suggests potential for improving knowledge in NCC as evidenced by the divergence in test scores between group 1 and group 2; and (3) wait-list control studies delivered over social media risk losing social media–based learners to follow-up.

Free, global, and easily accessible, Twitter has the ability to engage trainees and other healthcare providers from around the world and serve as a platform to provide medical education through many formats including journal clubs, conferences, and webinars.^5,8,9^ Our study was able to recruit learners from more than 22 countries (eTables 1 and 2, links.lww.com/NE9/A39) and from several medical specialties as well. This diverse group of learners participated in an innovative Tweetorial-based educational curriculum.

Tweetorial-based educational efforts lack data supporting their efficacy. The divergence in tests scores between our 2 groups from the preassessment to assessment 1 suggests knowledge improvements are possible, although our results should be interpreted cautiously in light of poor participant retention. A recent study of Tweetorials from the CardioNerds Academy analyzed 7 cardiology Tweetorials from 4 unique authors that found that the mean proportion of respondents felt comfortable with a given topic increased from 24.7% to 72.9% after completing a Tweetorial.^10^ Our study builds on this work by suggesting that knowledge may actually increase, a higher-order learning outcome.^11^

While both groups performed similarly on the baseline assessment, group 1 scored better on the second and third assessments, showing minimal knowledge decay. By contrast, group 2 did not seem to benefit from the curriculum, possibly due to poor engagement, evidenced by their Twitter metrics. A plausible explanation for a lower engagement rate in the second learner group is the methodology of wait-list–controlled studies that may lead to more attrition as other content vies for learner attention. Malcom Knowles' adult learning theory suggests that adults are most interested in learning that has immediate relevance and impact.^12^ The slow incremental deployment of our curriculum may have delayed its delivery to the point that group 2 learners had moved beyond the initial engagement on social media that inspired their participation. We had segmented the curriculum in this way to align with Mayer's principles of multimedia learning. According to Mayer,^13^ learners have limited capacity to their processing powers and learn better when messages are presented in user-paced segments rather than as a continuous unit. While this may be true, the peripatetic nature of residency training may demand that the subspecialized curriculum is delivered during the relevant clinical rotations lest all but the most intrinsically motivated learners be lost. Future studies of subspecialty education may need to account for this.

An alternative explanation would be that group 1 was inherently more motivated than group 2 and that this motivation could have led to group 1 learning more about NCC independent of our curriculum. The imbalance in trainee background is another possible explanation for the difference in performance between both learner groups because group 1 had more Neurology trainees compared with group 2, and the maturation effect is expected to be greater in Neurology trainees. Another possible explanation for the difference in performance between both learner groups is the presence of differential dropout, particularly given the considerable decrement in the number of participants completing all the assessments. Last, while NCC training is quite diverse throughout the world, we have no objective evidence to believe the country of origin or location of training played a substantial role in the score variation or engagement between both learner groups.

Twitter has emerged as an innovative platform that can effectively deliver educational content and engage diverse groups of healthcare professionals around the world. Our novel educational curriculum was able to reach learners from across 23 countries. On average, the learners accessed our educational material multiple times as measured by the number of engagements obtained via Twitter metrics. We followed expert advice in developing our Tweetorials, yet best practices for Tweetorials remain to be elucidated.^14,15^ Future research should focus on answering important questions to help optimize Tweetorial-based education, including how to maintain learner engagement through a longitudinal curriculum and how to assess outcomes. Our Tweetorial-based educational curriculum embraces the andragogy theory of adult education, which is based on self-directed, self-efficacious, problem-centered, independent and constructive methods.^16^ In addition, these microteaching threads are innovations in medical education because they are a novel teaching method that involve creation, integration, and application and thus merit inclusion within the Educational Scholarship through social media as defined by Holmboe et al.^17^

Because our Twitter profile (@NeurocriticalE) and curriculum was made available publicly in July 2022, it has garnered 1,395 followers, highlighting its expansive reach.

Our study has several limitations. There were more neurology trainees in group 1 than in group 2 despite randomization, although this difference was not statistically significant. There was a dramatic reduction in the number of participants who completed each assessment; however, the reductions were similar in each group. In addition, we were not able to assess Twitter metrics at an individual participant level, which would have allowed us to determine whether increased engagement with the curriculum led to more knowledge transfer. We also lacked qualitative feedback from the participants. Regarding our assessments, our multiple-choice–based assessments lacked measures of validity outside of internal content validity. We also lacked international diversity in the participating NCC faculty. Finally, our findings may have been biased toward more engagement with the platform because we recruited our learners on Twitter and they volunteered to take part. We do not know whether learners who do not already actively use Twitter would have interacted with our curriculum as favorably if assigned to it as part of a training program.

A Twitter-based educational curriculum disseminates teaching and improves knowledge in engaged learners. It is a low-resourced curriculum that worldwide neurosciences educators can design and adapt for their own learning needs and context. Furthermore, given the educational modality, worldwide collaborations and partnerships are possible in curriculum design, delivery, and evaluation. Twitter may be a valuable adjunct tool for learning and teaching in academia. Further research is necessary to discover best research designs for evaluating this platform. Best practices for consistent, longitudinal learner engagement also remain to be defined.

## Glossary

APP: advanced practice provider
NCC: neurocritical care
NCCU: NCC unit
NICU: neurologic intensive care unit
REDCap: Research Electronic Data Capture
